# Supplementary material for: Purchasing under threat: Changes in shopping patterns during the COVID-19 pandemic
Source: PLoS One. 2021 Jun 9;16(6):e0253231. doi: 10.1371/journal.pone.0253231 (PMC8189441; doi:10.1371/journal.pone.0253231)
Supplement: S1 Appendix — (DOCX) [file pone.0253231.s001.docx]

**S1 Appendix.**

**Fragebogen: Befragungen zu Auswirkungen der Corona-Pandemie auf das Kaufverhalten**

**Seite 1:**

**Einverständniserklärung**

Ich bin schriftlich über die Studie und den Studienablauf aufgeklärt worden. Ich habe die Teilnehmerinformation über die Untersuchung gelesen und verstanden. Sofern ich Fragen zu dieser Studie hatte, wurden sie vollständig und zu meiner Zufriedenheit beantwortet. Ich nehme an diese Studie freiwillig teil. Ich kann jederzeit ohne Angabe von Gründen meine Zustimmung zur Teilnahme widerrufen.

**Seite 2:**

1. Bitte geben Sie ihr Alter an. [Zahl]
2. Bitte geben Sie ihr Geschlecht an. [weiblich][männlich][divers]
3. In welchem Bundesland leben Sie? [Auswahl]
4. Besteht in Ihrem Wohnort aktuell Maskenpflicht beim Einkaufen? [ja] [nein] [weiß nicht]
5. Bitte geben Sie Ihren zuletzt erreichten Schulabschluss an. [kein Schulabschluss] [Hauptschulabschluss] [mittlere Reife] [Abitur] [Hoch-/ Fachhochschule]
6. Bitte geben Sie Ihren aktuellen Familienstand an. [ledig] [verheiratet] [geschieden] [verwitwet]
7. Wie viele Personen (inkl. Ihnen) leben in Ihrem Haushalt? [Zahl]

**Seite 3:**

1. Sind oder waren Sie, bestätigt durch einen positiven Test, an COVID-19 erkrankt? [ja] [nein]
2. Stehen Sie aktuell oder standen Sie aufgrund von COVID-19 unter Quarantäne? [ja] [nein]
3. Es zeigt sich ein erhöhtes Risiko für einen schweren Covid-19 Krankheitsverlauf ab einem Alter von 50-60 Jahren, für Raucher, für Personen mit Vorerkrankungen des Herzens, der Lunge, bei chronischen Lebererkrankungen, Diabetes mellitus, Krebserkrankungen oder einem geschwächten Immunsystem.
   Gehören Sie zu einer der oben genannten Risikogruppen? [ja] [nein]
4. Gehört eine Person aus Ihrem Haushalt oder eine Person mit der Sie regelmäßig Kontakt haben zur oben beschriebenen Risikogruppe? [ja] [nein]
5. Bitte geben Sie eine Einschätzung ab, wie oft Sie sich über COVID-19 informieren.
   Wie oft informieren Sie sich über den Verlauf von COVID-19? [Gar nicht] [Weniger als einmal am Tag] [Einmal am Tag] [Mehrmals täglich]

**Seite 4**

**Reguläres Einkaufsverhalten**

Im Folgenden stellen wir Ihnen einige Fragen zu Ihrem **regulären Einkaufsverhalten**. Die Fragen beziehen sich dabei auf den Kauf von **Lebensmitteln und Hygieneartikeln**. Denken Sie dabei an den **Januar 2020**, also an einen Zeitraum **vor dem akuten Ausbruch von COVID-19** in Deutschland.

1. Wer kaufte im Januar 2020 meistens für Sie ein?
   Mehrfachauswahl möglich. [Ich selbst] [Partner/in] [Familienmitglied] [andere]
2. Wo kauften Sie im Januar 2020 bevorzugt ein?
   Mehrfachauswahl möglich. [Supermarkt / Discounter] [Online] [Foodsharing] [andere]

**Veränderungen im Einkaufsverhalten**

Im Folgenden stellen wir Ihnen einige Fragen zu Veränderungen Ihres Einkaufsverhaltens. Die Fragen beziehen sich dabei wieder auf den Kauf von Lebensmitteln und Hygieneartikeln. Denken Sie dabei an den **März 2020**, also an einen Zeitraum während des akuten Ausbruchs von COVID-19 in Deutschland.

1. Wer kaufte im März 2020 meistens für Sie ein?
   Mehrfachauswahl möglich [Ich selbst] [Partner/in] [Familienmitglied] [andere]
2. Wo kauften Sie im März 2020 bevorzugt ein?
   Mehrfachauswahl möglich. [Supermarkt / Discounter] [Online] [Foodsharing] [andere]
3. **Verglichen mit Januar 2020, also vor dem Ausbruch der Corona-Pandemie in Deutschland, wie häufig waren Sie im März 2020 einkaufen?** **(*change in purchasing frequency*)**
    [deutlich seltener] [seltener] [etwas seltener] [genauso oft] [etwas häufiger] [häufiger] [deutlich häufiger]
4. **Verglichen mit Januar 2020, also vor dem Ausbruch der Corona-Pandemie in Deutschland, wie viele Produkte (Menge) haben Sie im März 2020 pro Einkauf eingekauft? (*change in purchasing quantity*)**  [deutlich weniger] [weniger] [etwas weniger] [genauso viel] [etwas mehr] [mehr] [deutlich mehr]

**Seite 5**

1. **Wie sehr hat sich die von Ihnen eingekaufte Menge der folgenden Produkte im Vergleich zu Januar 2020, also vor dem Ausbruch von COVID-19 in Deutschland, verändert? (*purchasing for quantity individual products*)**Konserven im **März** [deutlich weniger] [weniger] [etwas weniger] [genauso viel] [etwas mehr] [mehr] [deutlich mehr]

   Seife im **März** [deutlich weniger] [weniger] [etwas weniger] [genauso viel] [etwas mehr] [mehr] [deutlich mehr]

Toilettenpapier im **März** [deutlich weniger] [weniger] [etwas weniger] [genauso viel] [etwas mehr] [mehr] [deutlich mehr]

Nudeln/ Reis im **März** [deutlich weniger] [weniger] [etwas weniger] [genauso viel] [etwas mehr] [mehr] [deutlich mehr]

Hefe im **März** [deutlich weniger] [weniger] [etwas weniger] [genauso viel] [etwas mehr] [mehr] [deutlich mehr]

frische Produkte (z.B. Käse, Fleisch) im **März** [deutlich weniger] [weniger] [etwas weniger] [genauso viel] [etwas mehr] [mehr] [deutlich mehr]

Desinfektionsmittel im **März** [deutlich weniger] [weniger] [etwas weniger] [genauso viel] [etwas mehr] [mehr] [deutlich mehr]

**Seite 6**

1. **Das neuartige Coronavirus ist für mich… (*Perceived Threat of COVID-19*)**

Etwas, woran ich dauernd denke vs. etwas, woran ich fast nie denke [1-7]

Besorgniserregend vs. nicht besorgniserregend [1-7]

Angsteinflößend vs. nicht angsteinflößend [1-7]

Etwas, bei dem ich mich hilflos fühle vs. etwas, gegen das ich aktiv etwas tun kann [1-7]

Belastend vs. nicht belastend [1-7]

Nah vs. weit entfernt [1-7]

1. **Für wie wahrscheinlich halten Sie es, dass Sie sich beim Einkaufen mit COVID-19 anstecken?**

**[0-100] (*risk perception*)**

**Seite 7**

1. **Intolerance of Uncertainty Scale (12 Item Short Version)**

**Seite 8**

1. **STAI-Trait Scale**

***Anmerkung: Die folgenden Items wurden zur Erfassung sozialer Erwünschtheit (Satow, 2012) genutzt. Die Fragen wurden nicht (wie hier dargestellt) hintereinander präsentiert, sondern waren im Fragebogen verteilt.***

1. Ich habe schon mal Dinge weitererzählt, die ich besser für mich behalten hätte. [trifft gar nicht zu] [trifft eher nicht zu] [trifft eher zu] [trifft genau zu]
2. Im privaten Bereich habe ich schon mal Dinge gemacht, die besser nicht an die Öffentlichkeit kommen sollten. [trifft gar nicht zu] [trifft eher nicht zu] [trifft eher zu] [trifft genau zu]
3. Ich habe schon mal über andere gelästert oder schlecht über sie gedacht. [trifft gar nicht zu] [trifft eher nicht zu] [trifft eher zu] [trifft genau zu]
4. Ich habe schon mal etwas unterschlagen oder nicht gleich zurückgegeben. [trifft gar nicht zu] [trifft eher nicht zu] [trifft eher zu] [trifft genau zu]
5. Ich bilde mir meine Meinung immer sehr sorgfältig und würde niemals vorschnell urteilen. [trifft gar nicht zu] [trifft eher nicht zu] [trifft eher zu] [trifft genau zu]
6. Ich würde niemals schlecht über einen Kollegen oder meinen Arbeitgeber reden. [trifft gar nicht zu] [trifft eher nicht zu] [trifft eher zu] [trifft genau zu]
7. Ich würde mich niemals von einem Arzt krankschreiben lassen, ohne dass ich es wirklich bin. [trifft gar nicht zu] [trifft eher nicht zu] [trifft eher zu] [trifft genau zu]
