# Supplementary material for: Purchasing under threat: Changes in shopping patterns during the COVID-19 pandemic
Source: PLoS One. 2021 Jun 9;16(6):e0253231. doi: 10.1371/journal.pone.0253231 (PMC8189441; doi:10.1371/journal.pone.0253231)
Supplement: S2 Appendix — (DOCX) [file pone.0253231.s002.docx]

**S2 Appendix.**

**Questionnaire about the effects of the Coronavirus-Pandemic on consumer behavior**

**Page 1:**

**Informed Consent**

I have been informed about the study and its procedures. I have read and understood the participant information about the study. If I have had any questions about the study, they have been answered completely and to my full satisfaction. I voluntarily agree to take part in this study and I am free to withdraw at any time without giving a reason.

**Page 2:**

1. Please state your age. [number]
2. Please state your gender [female] [male] [other]
3. Which federal state do you live in? [selection]
4. In the state where you reside, is it mandatory to wear a face mask while grocery shopping? [yes] [no] [I don’t know]
5. Please indicate your highest academic degree. [no degree] [basic secondary schooling] [intermediate school certificate] [A-levels] [college-degree]
6. Please indicate your family status. [unmarried] [married] [divorced] [widowed]
7. How many people (including yourself) are living in your household? [number]

**Page 3:**

1. Are you or have you in the past been infected with COVID-19, confirmed by a positive test? [yes] [no]
2. Are you under quarantine or have you had to quarantine because of COVID-19? [yes] [no]
3. There is a higher risk for a serious course of COVID-19 for ages between 50-60 years, for smokers, for people with heart disease, lung disease, chronic liver disease, Diabetes mellitus, cancer, or an impaired immune system.
   Do you belong to one of these groups? [yes] [no]
4. Does a household member or another person you have frequent contact with belong to one of the above stated groups? [yes] [no]
5. Please estimate how often you inform yourself about COVID-19.
   How often do you inform yourself about the development of COVID-19? [never] [less than once a day] [once a day] [multiple times a day]

**Page 4**

**Regular shopping behavior**

In the following we will ask you questions about your **typical shopping behavior**. These questions relate to the purchase of **groceries and sanitary products**. To answer these questions, please recall **January 2020**, before the Coronavirus pandemic began in Germany.

1. Who went grocery shopping for you in January 2020?
   You may choose more than one answer. [myself] [partner] [family member] [others]
2. Where did you prefer to go grocery shopping in January 2020?
   You may choose more than one answer. [supermarket/ discounter] [online] [foodsharing] [others]

**Changes in shopping behavior**

In the following we will ask you questions about changes in your shopping behavior. The questions again relate to the purchase of groceries and sanitary products. To answer these questions, please recall **March 2020**, when the Coronavirus began spreading in Germany.

1. Who went grocery shopping for you in March 2020?
   You may choose more than one answer. [myself] [partner] [family member] [others]
2. Where did you prefer to go grocery shopping in March 2020?
   You may choose more than one answer. [supermarket/ discounter] [online] [foodsharing] [others]
3. **Compared to January 2020, before the outbreak of the Corona pandemic in Germany, how often did you go shopping in March 2020?** **(*change in purchasing frequency*)**
   [much less frequently] [less frequently] [little less frequently] [just as often] [more frequently] [much more frequently]
4. **Compared to January 2020, before the outbreak of the Corona pandemic in Germany, how much (quantity) did you buy per purchase in March 2020?** **(*change in purchasing quantity*)** [much less] [less] [a little less] [just as much] [a little more] [more] [much more]

**Seite 5**

1. **How much has the amount of purchased products changed compared to January 2020, before the Coronavirus-Pandemic began?** **(*purchasing for quantity individual products*)**

   Canned goods in **March** [much less] [less] [somewhat less] [just as much] [a little more][more] [much more]

   Soap in **March** [much less] [less] [a little less] [just as much] [a little more] [more] [much more]

   Toilet paper in **March** [much less] [less] [a little less] [just as much] [a little more] [more] [much more]

   Pasta / Rice in **March** [much less] [less] [a little less] [just as much] [a little more] [more] [much more]

   Yeast in **March** [much less] [less] [a little less] [just as much] [a little more] [more] [much more]

Fresh produce (such as cheese, meat) in **March** [much less] [less] [a little less] [just as much] [a little more] [more] [much more]

Disinfectant in **March** [much less] [less] [a little less] [just as much] [a little more] [more] [much more]

**Page 6**

1. **For me the novel Coronavirus is…** **(*Perceived Threat of COVID-19*)**
   Something that I think about constantly vs. something that I rarely ever think about [1-7]

   Concerning vs. not concerning [1-7]

   Fear-inducing vs. not fear-inducing [1-7]

   Something that I feel helpless about vs. something that I can do something about [1-7]

   Stressful vs. not stressful [1-7]

   Far vs. close [1-7]
2. **How likely do you think it is that** **You will get infected with COVID-19 while grocery shopping? [0-100]** **(*risk perception*)**

**Page 7**

1. **Intolerance of Uncertainty Scale (12 Item short version)**

**Page 8**

1. **STAI-Trait Scale**

***Note: The following Items were used to capture social desirability (Satow, 2012). The questions were not asked directly one after the other (as shown here), but were distributed throughout questionnaire***

1. I have passed on things that I should have kept to myself. [not true at all] [not really true] [probably true] [completely true]
2. In private settings I have done things that should not be brought to light. [not true at all] [not really true] [probably true] [completely true]
3. I have gossiped or thought badly of someone before. [not true at all] [not really true] [probably true] [completely true]
4. I have withheld something before or have not given something back straight away. [not true at all] [not really true] [probably true] [completely true]
5. I always form my opinion with careful consideration and would never judge prematurely. [not true at all] [not really true] [probably true] [completely true]
6. I would never talk badly about a colleague or my boss. [not true at all] [not really true] [probably true] [completely true]
7. I would never take a sick leave without actually being sick. [not true at all] [not really true] [probably true] [completely true]
