# Supplementary material for: Purchasing under threat: Changes in shopping patterns during the COVID-19 pandemic
Source: PLoS One. 2021 Jun 9;16(6):e0253231. doi: 10.1371/journal.pone.0253231 (PMC8189441; doi:10.1371/journal.pone.0253231)
Supplement: S3 Appendix — (DOCX) [file pone.0253231.s003.docx]

**S3 Appendix. Non-parametric Data Analysis**

The following tables (Table 1 -3) provide non-parametric analyses of the data. Note that the obtained results are highly comparable to the parametric solutions.

|  | **Change in Purchasing Frequency** | | | **Change in Purchasing Quantity** | | |
| --- | --- | --- | --- | --- | --- | --- |
| *Predictors* | *Odds Ratios* | *CI* | *p-value* | *Odds Ratios* | *CI* | *p-value* |
| Sex | **1.59** | **1.11 – 2.28** | **0.012** | 0.75 | 0.51 – 1.08 | 0.127 |
| Age | 0.96 | 0.83 – 1.12 | 0.625 | 0.77 | 0.65 – 0.90 | **0.002** |
| Educational Level | **0.78** | **0.67 – 0.90** | **0.001** | **1.28** | **1.10 – 1.50** | **0.002** |
| Household Size | 0.99 | 0.86 – 1.15 | 0.882 | 1.00 | 0.85 – 1.16 | 0.981 |
| Social Desirability Bias | 1.01 | 0.87 – 1.17 | 0.903 | 0.92 | 0.78 – 1.07 | 0.270 |
| Threat of COVID-19 | **0.59** | **0.49 – 0.70** | **<0.001** | **1.55** | **1.29 – 1.87** | **<0.001** |
| IUS | 1.13 | 0.94 – 1.36 | 0.212 | 1.01 | 0.84 – 1.23 | 0.881 |
| Trait-Anxiety | 0.92 | 0.77 – 1.10 | 0.373 | 1.02 | 0.84 – 1.24 | 0.821 |
| Media Exposure | **0.79** | **0.67 – 0.92** | **0.004** | **1.36** | **1.14 – 1.61** | **0.001** |
| Perceived Risk | **0.81** | **0.70 – 0.94** | **0.006** | **1.27** | **1.08 – 1.49** | **0.003** |
| Observations | 678 | | | 678 | | |
| R^2^ Nagelkerke | 0.168 | | | 0.165 | | |

**Table 1. Overall Model: Prediction of change in purchasing behavior (frequency/quantity) using Ordinal Logistic Regression. Compare with Table 3 in manuscript.**

Significant odds ratios (p < .05) of the ordinal logistic regression analysis are printed in bold. Coding purchasing quantity: 0 = same, 1 = a litte more, 2 = more, 3 = much more. Coding for purchasing frequency:

0 = same, -1 = a little less frequently, -2 = less frequently, -3 = much less frequently. IUS = Intolerance of Uncertainty. Dichotomous Variables: Coding for sex: female = 0, male = 1; coding for being part a risk group for a severe COVID-19 disease course: no = 0, yes = 1.

**Table 2. Prediction of change in purchasing quantity for individual products. Compare with S4 Table.**

|  | **Non-perishable Food** | | | **Hygiene Products** | | | **Fresh Food** | | |
| --- | --- | --- | --- | --- | --- | --- | --- | --- | --- |
| *Predictors* | *Odds Ratios* | *95% CI* | *p-value* | *Odds Ratios* | *95% CI* | *p-value* | *Odds Ratios* | *95% CI* | *p-value* |
| Sex | 1.17 | 0.80 – 1.71 | 0.424 | 1.05 | 0.73 – 1.52 | 0.788 | 1.39 | 0.88 – 2.17 | 0.148 |
| Age | **1.25** | **1.04 – 1.51** | **0.021** | **0.77** | **0.64 – 0.92** | **0.005** | **0.73** | **0.58 – 0.92** | **0.008** |
| Educational Level | 1.04 | 0.89 – 1.22 | 0.608 | **1.17** | **1.00 – 1.37** | **0.048** | 1.07 | 0.88 – 1.31 | 0.505 |
| Household Size | 1.07 | 0.92 – 1.25 | 0.367 | 0.96 | 0.82 – 1.12 | 0.621 | 0.92 | 0.73 – 1.13 | 0.447 |
| Social Desirability Bias | **0.84** | **0.71 – 0.98** | **0.028** | 0.86 | 0.74 – 1.00 | 0.056 | 0.86 | 0.71 – 1.04 | 0.127 |
| Risk Group (self) | 1.10 | 0.75 – 1.60 | 0.624 | 0.93 | 0.65 – 1.34 | 0.695 | 1.10 | 0.70 – 1.74 | 0.670 |
| Risk Group (others) | 1.16 | 0.84 – 1.62 | 0.371 | 0.97 | 0.70 – 1.33 | 0.834 | 1.41 | 0.95 – 2.12 | 0.092 |
| Media Exposure | **1.30** | **1.10 – 1.55** | **0.002** | **1.36** | **1.15 – 1.61** | **<0.001** | 1.16 | 0.94 – 1.43 | 0.173 |
| Threat of COVID-19 | **1.81** | **1.51 – 2.19** | **<0.001** | **1.39** | **1.17 – 1.66** | **<0.001** | **1.34** | **1.07 – 1.68** | **0.010** |
| Risk Perception | **1.20** | **1.02 – 1.42** | **0.027** | 1.15 | 0.98 – 1.35 | 0.092 | 1.02 | 0.83 – 1.24 | 0.860 |
| Intolerance of Uncertainty | 1.14 | 0.97 – 1.35 | 0.106 | **1.28** | **1.09 – 1.51** | **0.003** | 1.22 | 1.00 – 1.49 | 0.055 |
| Observations | 789 | | | 801 | | | 749 | | |
| R^2^ Tjur | 0.146 | | | 0.118 | | | 0.055 | | |

Significant odds ratios (p < .05) of the logistic regression analysis are printed in bold. Coding for each product category: 0 = same, 1 = more.

Dichotomous Variables: Coding for sex: female = 0, male = 1; coding for being part a risk group for a severe COVID-19 disease course: no = 0, yes = 1.

|  | *Change in Purchasing Frequency* | *Change in Purchasing Quantity* | *Sex* | *Age* | *Educational Level* | *Household Size* | *SDB* | *Threat of COVID-19* | *Risk Perception* | *IUS* | *STAI* | *Media Exposure* | *Risk Self* |
| --- | --- | --- | --- | --- | --- | --- | --- | --- | --- | --- | --- | --- | --- |
| *Change in*  *Purchasing*  *Quantity* | -.592^***^ |  |  |  |  |  |  |  |  |  |  |  |  |
| *Sex* | .139^***^ | -.085^*^ |  |  |  |  |  |  |  |  |  |  |  |
| *Age* | -.020 | -.118^**^ | -.048 |  |  |  |  |  |  |  |  |  |  |
| *Educational Level* | -.130^***^ | .132^***^ | .021 | -.064 |  |  |  |  |  |  |  |  |  |
| *Householdsize* | -.020 | .044 | -.017 | -.132^***^ | .020 |  |  |  |  |  |  |  |  |
| *SDB* | .005 | -.064 | -.098^*^ | .151^***^ | -.006 | -.012 |  |  |  |  |  |  |  |
| *Threat of*  *COVID-19* | -.334^***^ | .313^***^ | -.153^***^ | -.102^**^ | .072 | .047 | -.088^*^ |  |  |  |  |  |  |
| *Risk Perception* | -.217^***^ | .224^***^ | -.052 | -.095^*^ | -.001 | .051 | -.104^**^ | .401^***^ |  |  |  |  |  |
| *IUS* | -.083^*^ | .137^***^ | -.047 | -.193^***^ | -.057 | .008 | -.126^**^ | .314^***^ | .203^***^ |  |  |  |  |
| *STAI* | -.082^*^ | .109^**^ | -.051 | -.232^***^ | -.101^**^ | -.011 | -.228^***^ | .307^***^ | .224^***^ | .586^***^ |  |  |  |
| *Media*  *Exposure* | -.165^***^ | .143^***^ | .006 | .284^***^ | -.016 | .016 | .106^**^ | .224^***^ | -.002 | .060 | -.088^*^ |  |  |
| *Risk Self* | -.053 | -.058 | .006 | .460^***^ | -.102^**^ | -.156^***^ | .003 | .038 | .083^*^ | -.066 | -.018 | .112^**^ |  |
| *Risk Loved* | -.066 | -.019 | -.076^*^ | .145^***^ | -.027 | .041 | -.017 | .030 | .012 | -.033 | .009 | .027 | .354^***^ |

**Table 3. Bivariate Correlation (Spearman´s rank correlation coefficient) between variables. Compare to S1 Table in the manuscripts.**

*N =* 678. Significant correlations (Spearman´s rho) are printed in black. Coding for dichotomous variables: Risk Group (0 = No, 1 = Yes), Sex (0 = female, 1 = male). SDB = Social Desirability Bias; IUS = Intolerance of Uncertainty; STAI = Trait Anxiety. **p<.05, **p<.01, ***p<.001*
