# Supplementary material for: Purchasing under threat: Changes in shopping patterns during the COVID-19 pandemic
Source: PLoS One. 2021 Jun 9;16(6):e0253231. doi: 10.1371/journal.pone.0253231 (PMC8189441; doi:10.1371/journal.pone.0253231)
Supplement: S1 Table — (DOCX) [file pone.0253231.s004.docx]

|  | *Change in Purchasing Frequency* | *Change in Purchasing Quantity* | *Sex* | *Age* | *Educational Level* | *Household Size* | *SDB* | *Threat of COVID-19* | *Risk Perception* | *IUS* | *STAI* | *Media Exposure* | *Risk Self* |
| --- | --- | --- | --- | --- | --- | --- | --- | --- | --- | --- | --- | --- | --- |
| *Change in*  *Purchasing*  *Quantity* | -.607^***^ |  |  |  |  |  |  |  |  |  |  |  |  |
| *Sex* | .129^***^ | -.068 |  |  |  |  |  |  |  |  |  |  |  |
| *Age* | -.033 | -.108^**^ | -.051 |  |  |  |  |  |  |  |  |  |  |
| *Educational Level* | -.127^***^ | .122^**^ | .017 | -.103^**^ |  |  |  |  |  |  |  |  |  |
| *Householdsize* | -.018 | .038 | -.026 | -.077^*^ | .035 |  |  |  |  |  |  |  |  |
| *SDB* | .007 | -.049 | -.111^**^ | .159^***^ | -.011 | -.013 |  |  |  |  |  |  |  |
| *Threat of*  *COVID-19* | -.319^***^ | .314^***^ | -.156^***^ | -.104^**^ | .086^*^ | .017 | -.095^*^ |  |  |  |  |  |  |
| *Risk Perception* | -.189^***^ | .203^***^ | -.048 | -.068 | -.018 | .001 | -.068 | .358^***^ |  |  |  |  |  |
| *IUS* | -.074 | .121^**^ | -.051 | -.188^***^ | -.038 | -.028 | -.133^***^ | .322^***^ | .208^***^ |  |  |  |  |
| *STAI* | -.082^*^ | .111^**^ | -.043 | -.230^***^ | -.080^*^ | -.036 | -.223^***^ | .306^***^ | .218^***^ | .600^***^ |  |  |  |
| *Media*  *Exposure* | -.174^***^ | .146^***^ | -.007 | .273^***^ | -.015 | .031 | .106^**^ | .238^***^ | .025 | .063 | -.088^*^ |  |  |
| *Risk Self* | -.066 | -.047 | .006 | .464^***^ | -.119^**^ | -.125^**^ | .013 | .032 | .087^*^ | -.063 | -.009 | .105^**^ |  |
| *Risk Loved* | -.069 | -.012 | -.076^*^ | .147^***^ | -.026 | .015 | -.023 | .028 | .001 | -.038 | .006 | .026 | .354^***^ |

**S1 Table. Bivariate Correlations between variables.**

*N =* 678. Significant correlations (Pearson´s r) are printed in black. Coding for dichotomous variables: Risk Group (0 = No, 1 = Yes), Sex (0 = female, 1 = male). SDB = Social Desirability Bias; IUS = Intolerance of Uncertainty; STAI = Trait Anxiety. **p<.05, **p<.01, ***p<.001*
