# Supplementary material for: Purchasing under threat: Changes in shopping patterns during the COVID-19 pandemic
Source: PLoS One. 2021 Jun 9;16(6):e0253231. doi: 10.1371/journal.pone.0253231 (PMC8189441; doi:10.1371/journal.pone.0253231)
Supplement: S2 Table — (DOCX) [file pone.0253231.s005.docx]

|  | *Change in Purchasing Frequency* | *Change in Purchasing Quantity* | *Sex* | *Age* | *Educational Level* | *Householdsize* | *SDB* | *Threat of COVID-19* | *Risk Perception* | *IUS* | *STAI* | *Media Exposure* | *Risk Self* |
| --- | --- | --- | --- | --- | --- | --- | --- | --- | --- | --- | --- | --- | --- |
| *Change in Purchasing Quantity* | -.309^***^ |  |  |  |  |  |  |  |  |  |  |  |  |
| *Sex* | .107^**^ | -.035 |  |  |  |  |  |  |  |  |  |  |  |
| *Age* | -.037 | -.121^***^ | -.045 |  |  |  |  |  |  |  |  |  |  |
| *Educational Level* | -.058 | .095^**^ | .002 | -.099^**^ |  |  |  |  |  |  |  |  |  |
| *Householdsize* | -.002 | .022 | -.047 | -.097^**^ | .039 |  |  |  |  |  |  |  |  |
| *SDB* | -.014 | -.081^*^ | -.113^**^ | .179^***^ | .001 | -.016 |  |  |  |  |  |  |  |
| *Threat of COVID-19* | -.184^***^ | .226^***^ | -.150^***^ | -.110^**^ | .116^***^ | .024 | -.083^*^ |  |  |  |  |  |  |
| *Risk Perception* | -.122^***^ | .141^***^ | -.036 | -.083^*^ | -.026 | -.038 | -.088^*^ | .343^***^ |  |  |  |  |  |
| *IUS* | .018 | .149^***^ | -.033 | -.179^***^ | -.033 | -.026 | -.136^***^ | .300^***^ | .237^***^ |  |  |  |  |
| *STAI* | -.021 | .109^**^ | -.043 | -.231^***^ | -.048 | -.006 | -.224^***^ | .325^***^ | .232^***^ | .605^***^ |  |  |  |
| *Media Exposure* | -.060 | .087^*^ | -.006 | .269^***^ | -.025 | .039 | .092^**^ | .241^***^ | .033 | .067 | -.059 |  |  |
| *Risk Self* | -.059 | -.094^**^ | -.001 | .438^***^ | -.119^***^ | -.134^***^ | .010 | .048 | .106^**^ | -.042 | .011 | .090^*^ |  |
| *Risk Loved* | -.064 | -.004 | -.078^*^ | .152^***^ | -.033 | -.017 | -.039 | .042 | .008 | -.022 | .019 | .024 | .372^***^ |

**S2 Table. Bivariate correlations between variables for the full range scale.**

*N* = 813. Significant correlations are printed in black. Coding for dichotomous variables: Risk Group (0 = No, 1 = Yes), Sex (0 = female, 1 = male). SDB = Social Desirability Bias; IUS = Intolerance of Uncertainty; STAI = Trait Anxiety. **p<.05, **p<.01, ***p<.001*
