# Supplementary material for: Purchasing under threat: Changes in shopping patterns during the COVID-19 pandemic
Source: PLoS One. 2021 Jun 9;16(6):e0253231. doi: 10.1371/journal.pone.0253231 (PMC8189441; doi:10.1371/journal.pone.0253231)
Supplement: S3 Table — (DOCX) [file pone.0253231.s006.docx]

|  | **Change in Purchasing Frequency** | | | **Change in Purchasing Quantity** | | |
| --- | --- | --- | --- | --- | --- | --- |
| *Predictors* | *b* | *t-statistic* | *p-value* | *b* | *t-statistic* | *p-value* |
| **Baseline Model** |  |  |  |  |  |  |
| Sex | **.26** | **3.01** | **.003** | -.11 | 1.34 | .181 |
| Age | -.04 | 1.09 | .278 | **-.10** | **2.87** | **.004** |
| Educational Level | -.06 | 1.76 | .079 | **.09** | **2.44** | **.015** |
| Householdsize | .00 | 0.04 | .969 | .01 | 0.16 | .876 |
| Social Desiability Bias | .01 | 0.14 | .888 | -.07 | 1.93 | .055 |
| **Added Predictor** |  |  |  |  |  |  |
| Threat of COVID-19 | **-.17** | **4.92** | **<.001** | **.20** | **5.82** | **<.001** |
| Intolerance of Uncertainty | .01 | 0.37 | .708 | **.13** | **3.67** | **<.001** |
| Trait Anxiety | -.03 | 0.82 | .415 | **.08** | **2.21** | **.028** |
| Risk Group (Self) | -.13 | 1.58 | .114 | -.10 | 1.23 | .218 |
| Risk Group (Loved) | -.11 | 1.50 | .135 | .02 | 0.23 | .818 |
| Media Exposure | -.06 | 1.53 | .126 | **.13** | **3.69** | **<.001** |
| Risk Perception | **-.13** | **3.58** | **<.001** | **.13** | **3.72** | **<.001** |

**S3 Table.** **Prediction of change in purchasing behavior (full range scale).**

*N = 813*. Significant regression weights (*p < .05*) of the multiple regression analysis are printed in bold. All continuous variables were included as z-standardized variables. Dichotomous Variables: Coding for sex: female = 0, male = 1; coding for being part a risk group for a severe COVID-19 disease course: no = 0, yes = 1. COVID-19 Threat was the best predictor for change in purchasing frequency

(*R²adj. = .04*) and change in purchasing quantity (*R²adj. = .06*).
