# Supplementary material for: Purchasing under threat: Changes in shopping patterns during the COVID-19 pandemic
Source: PLoS One. 2021 Jun 9;16(6):e0253231. doi: 10.1371/journal.pone.0253231 (PMC8189441; doi:10.1371/journal.pone.0253231)
Supplement: S4 Table — (DOCX) [file pone.0253231.s007.docx]

**S4 Table. Multiple Regression Analysis for change in purchasing quantity controlling for purchasing frequency.**

|  | **Change in Purchasing Quantity** | | |
| --- | --- | --- | --- |
| *Predictors* | *b* | *t-Statistic* | *p-value* |
| FreqMarch | **-0.56** | **-17.36** | **<0.001** |
| Sex | 0.03 | 0.41 | 0.681 |
| Age | -0.12 | -3.72 | **<0.001** |
| Education | 0.03 | 1.07 | 0.287 |
| Householdsize | 0.01 | 0.47 | 0.636 |
| Social Desirability | -0.02 | -0.58 | 0.561 |
| Perceived Threat of COVID-19 | **0.08** | **2.22** | **0.026** |
| IUS | 0.02 | 0.50 | 0.619 |
| STAI | -0.01 | -0.17 | 0.864 |
| Media Exposure | 0.06 | 1.85 | 0.065 |
| Risk Perception | 0.06 | 1.72 | 0.086 |
| Observations | 678 | | |
| R^2^ / R^2^adjusted | 0.405 / 0.395 | | |
