# Supplementary material for: Purchasing under threat: Changes in shopping patterns during the COVID-19 pandemic
Source: PLoS One. 2021 Jun 9;16(6):e0253231. doi: 10.1371/journal.pone.0253231 (PMC8189441; doi:10.1371/journal.pone.0253231)
Supplement: S5 Table — (DOCX) [file pone.0253231.s008.docx]

**S5 Table. Prediction of change in purchasing quantity for individual products.**

Significant regression weights (p < .05) of the multiple regression analysis are printed in bold. All continuous variables were included as z-standardized variables.

Dichotomous Variables: Coding for sex: female = 0, male = 1; coding for being part a risk group for a severe COVID-19 disease course: no = 0, yes = 1.

|  | **NonPerishableFood** | | | **HygieneProducts** | | | **FreshFood** | | |
| --- | --- | --- | --- | --- | --- | --- | --- | --- | --- |
| *Predictors* | *b* | *95% CI* | *p* | *b* | *95% CI* | *p* | *b* | *95% CI* | *p* |
| Sex | .04 | -.12 – .20 | .631 | .11 | -.06 – .27 | .199 | .13 | -.05 – .31 | .149 |
| Age | **.09** | **.01 – .17** | **.022** | -.01 | -.09 – .07 | .762 | -.08 | -.16 – .01 | .084 |
| Educational Level | .05 | -.02 – .11 | .172 | .06 | -.00 – .13 | .069 | -.00 | -.07 – .07 | .982 |
| Household Size | .02 | -.04 – .09 | .522 | -.02 | -.08 – .05 | .603 | -.02 | -.09 – .05 | .607 |
| Social Desirability Bias | **-.08** | **-.15 – -.02** | **.016** | -.06 | -.12 – .01 | .103 | -.03 | -.10 – .05 | .470 |
| Risk Group (self) | .02 | -.14 – .18 | .769 | -.09 | -.25 – .07 | .284 | .02 | -.16 – .19 | .836 |
| Risk Group (others) | **.17** | **.03 – .31** | **.020** | .09 | -.06 – .23 | .241 | .12 | -.03 – .27 | .125 |
| Media Exposure | **.11** | **.04 – .18** | **.003** | **.13** | **.05 – .20** | **.001** | .05 | -.03 – .13 | .195 |
| Perceived Threat of COVID-19 | **.21** | **.14 – .29** | **<.001** | **.17** | **.09 – .25** | **<.001** | **.10** | **.01 – .18** | **.023** |
| Risk Perception | **.11** | **.04 – .18** | **.003** | .06 | -.01 – .14 | .077 | .02 | -.06 – .10 | .609 |
| Intolerance of Uncertainty | **.10** | **.03 – .17** | **.006** | **.14** | **.07 – .21** | **<.001** | **.09** | **.01 – .16** | **.028** |
| Observations | 789 | | | 801 | | | 749 | | |
| R^2^ / R^2^ adjusted | 0.154 / 0.142 | | | 0.121 / 0.109 | | | 0.044 / 0.029 | | |
