# Supplementary material for: Purchasing under threat: Changes in shopping patterns during the COVID-19 pandemic
Source: PLoS One. 2021 Jun 9;16(6):e0253231. doi: 10.1371/journal.pone.0253231 (PMC8189441; doi:10.1371/journal.pone.0253231)
Supplement: S6 Table — (DOCX) [file pone.0253231.s009.docx]

|  | **Non-perishable Food** | | | **Hygiene Products** | | | **Fresh Food** | | |
| --- | --- | --- | --- | --- | --- | --- | --- | --- | --- |
| *Predictors* | *b* | *95% CI* | *p* | *b* | *95% CI* | *p* | *b* | *95% CI* | *p* |
| Sex | .07 | -.06 – .20 | .261 | .08 | -.04 – .20 | .204 | .09 | -.03 – .21 | .156 |
| Age | .00 | -.00 – .00 | .729 | -.00 | -.01 – .00 | .153 | -.00 | -.00 – .00 | .640 |
| Educational Level | .04 | -.02 – .09 | .223 | **.07** | **.01 – .12** | **.018** | .01 | -.04 – .07 | .639 |
| Household Size | .02 | -.02 – .05 | .281 | .00 | -.03 – .03 | .964 | .01 | -.02 – .04 | .668 |
| Social Desirability Bias | **-.03** | **-.05 – -.00** | **.026** | -.02 | -.04 – .00 | .100 | -.01 | -.03 – .01 | .482 |
| Risk Group (self) | .05 | -.08 – .18 | .463 | -.06 | -.19 – .06 | .308 | -.02 | -.14 – .10 | .712 |
| Risk Group (others) | -.04 | -.15 – .08 | .518 | .02 | -.09 – .13 | .714 | .05 | -.05 – .16 | .338 |
| Media Exposure | .07 | -.00 – .15 | .065 | **.10** | **.02 – .17** | **.010** | .03 | -.05 – .10 | .454 |
| Perceived Threat of COVID-19 | **.10** | **.05 – .14** | **<.001** | **.07** | **.03 – .12** | **.001** | .02 | -.02 – .06 | .275 |
| Risk Perception | **.00** | **.00 – .00** | **.036** | .00 | -.00 – .00 | .145 | .00 | -.00 – .00 | .662 |
| Intolerance of Uncertainty | **.01** | **.00 – .01** | **.003** | **.01** | **.00 – .01** | **.001** | .00 | -.00 – .01 | .095 |
| Observations | 810 | | | 813 | | | 794 | | |
| R^2^ / R^2^ adjusted | 0.096 / 0.083 | | | 0.096 / 0.083 | | | 0.017 / 0.003 | | |

**S6 Table. Prediction of change in purchasing quantity for individual products (full range scale).**

Significant regression weights (p < .05) of the multiple regression analysis are printed in bold. All continuous variables were included as z-standardized variables.

Dichotomous Variables: Coding for sex: female = 0, male = 1; coding for being part a risk group for a severe COVID-19 disease course: no = 0, yes = 1.
