# Supplementary material for: Purchasing under threat: Changes in shopping patterns during the COVID-19 pandemic
Source: PLoS One. 2021 Jun 9;16(6):e0253231. doi: 10.1371/journal.pone.0253231 (PMC8189441; doi:10.1371/journal.pone.0253231)
Supplement: S7 Table — (DOCX) [file pone.0253231.s010.docx]

**S7 Table. Predictors of Perceived Threat of COVID-19.**

| *Predictors* | *b* | *95% CI* | *p* |
| --- | --- | --- | --- |
| Sex | **-0.33** | **-0.47 – -0.18** | **<0.001** |
| Age | **-0.09** | **-0.15 – -0.02** | **0.008** |
| Educational Level | **0.13** | **0.08 – 0.19** | **<0.001** |
| Household Size | 0.01 | -0.05 – 0.07 | 0.839 |
| Social Desirability Bias | -0.03 | -0.09 – 0.03 | 0.379 |
| Risk Perception | **0.26** | **0.20 – 0.32** | **<0.001** |
| Intolerance of Uncertainty | 0.07 | -0.00 – 0.15 | 0.050 |
| Trait-Anxiety | **0.21** | **0.13 – 0.29** | **<0.001** |
| Media Exposure | **0.27** | **0.21 – 0.33** | **<0.001** |

*N = 813*. Continuous variables were included as z-standardized variables. Coding for sex: female = 0, male = 1. The model explained 29.1% (*R²adj = .283*) of the variance of *Perceived Threat of COVID-19*.
