# Supplementary material for: Purchasing under threat: Changes in shopping patterns during the COVID-19 pandemic
Source: PLoS One. 2021 Jun 9;16(6):e0253231. doi: 10.1371/journal.pone.0253231 (PMC8189441; doi:10.1371/journal.pone.0253231)
Supplement: Attachment — Submitted filename: Response to Reviewers.docx [file pone.0253231.s011.docx]

**Response to Reviewers**

Dear Dr. Andreatta,

First of all, we would like to thank you and the reviewers for the helpful and valuable feedback on the manuscript. We were pleased with the overall positive tone of the reviews. Although the reviewers found the study to be interesting and overall well done, they also raised a number of issues that need some clarification. Below we provide detailed responses to the reviewer's comments.

**Reviewer 1**

**In this study, the authors investigated changes in purchasing patterns during the first wave of the COVID-19 pandemic in Germany in a retrospective online survey. Results show that high levels of perceived COVID-19-threat, intolerance of uncertainty and media exposure were associated with reduced purchase frequency and increased number of products bought. Moreover, results have been linked to animal models of foraging behavior under threat.
The study background, procedure, results and discussion are concise and clear; data are freely available. The results of the present survey are of broad interest. I do have only some minor suggestions.**

**Title: I would recommend a more eye-catching title avoiding repetition of “purchasing” (e.g., Shopping or buying)**

In accordance with the reviewer’s suggestion we changed the title to “Purchasing under threat: changes in shopping patterns during the COVID-19 pandemic”.

**Introduction: Please cite international data for “worldwide panic buying” (p. 3, line 1).**

We thank the reviewer for raising our attention to this point. In accordance with the reviewer’s suggestion we now additionally cite the international data, e.g., data from USA and UK.

**Introduction:**

“The spreading of the coronavirus disease (COVID-19) has led to worldwide stockpiling of food and hygiene products which caused temporally shortages [1]. In early March 2020, when the number of daily COVID-19 infections reached its peak in Germany [2], the German Federal Statistical Office recorded an enormous increase in sales of goods of sanitary and daily needs [3]: e.g., early in March 2020, a 150% increase for pasta, 153% for soap, and 751% for disinfectants. Similar changes in shopping behavior were recorded in the USA [4] and the UK [5]. At the same time, studies indicated an increase in fear and worries related to the virus [6, 7]. […]”

**Please provide more detailed information on the online assessment method used (software; e.g. EvaSys; SociSurvey).**

In accordance with the reviewer’s suggestion, we added more detailed information as follows:

“The online questionnaire was realized using SoSci Survey [22] and was published on soscisurvey.de.”

**I would stick to the previous wording “Perceived Threat of COVID-19 in the whole manuscript (p. 6, Predictors […])**

In accordance with the reviewer’s suggestion, we now use the wording “Perceived Threat of COVID-19” in the whole manuscript.

**p. 7, line 14f: description of the Likert scale has no quotation marks (unlike the previous scales), see also Demographic Variables below (I would stick to one formatting)**

In accordance with the reviewer’s suggestion, we now use quotation marks when citing scale levels throughout the manuscript.

**Methods: p. 4: May_18th (missing space)**

**Results: p.16, line 9 and p. 14, lines 23 and 25 and p. 15 lines 1 and8: p <_.001 (missing spaces)**

We thank the author for raising our attention to this point. We checked and corrected the manuscript for missing spaces.

**Discussion: I would recommend including further research in a more detailed discussion. For example, Leung et al. (2021) recently published an interesting analysis on social media content, highlighting the role of social media in anxiety and panic buying behaviors (see p.17, line 4f). Also, there is a publication by Bentall et al. (2021) on pandemic buying which is also based on animal foraging theory, which should be included. (see also Lehberger et al., 2021; Micalizzi et al, 2020).**

In accordance with the reviewer´s suggestion we now provide a more detailed discussion, referencing to the suggested literature.

“At the same time, media exposure was associated with an increase in purchasing quantity and a decrease in purchasing frequency. Another study, using structural equation models, revealed that cyberchondria - that is, excessive information gathering about COVID-19 combined with feelings of frustration and anxiety - is positively associated with the intention to make unusual purchases [38]. A qualitative study on contents on twitter about toilet paper hoarding found out that nearly half of the analyzed tweets expressed negative feelings toward panic buying [39]. The authors hypothesize that this might lead to emotional distress, depression and anxiety-driven panic buying (see also [40]).”

**When targeting the “Threat of COVID-19”, it would be interesting whether reducing the number (or encounter probability) of “predators” (which is, other potentially contagious individuals in the human model) in the supermarket might reduce perceived threat. Is there any animal research paralleling this restriction measure (already taken by several countries)?**

We thank the reviewer for raising our attention to this interesting aspect. There is animal research indicating that foraging behavior in rodents varies as a function of shock density (Helmstetter & Fanselow, 1993). Low shock density was associated with an increase in meal size (quantity) but only little change in meal frequency. Higher levels of shock were associated with an increase in meal size while reducing frequency to avoid shock. As hypothesized by the reviewer, this finding indicates that encounter probability influences the extend of change in foraging behavior. From this point of view, one might expect that the introduction of restriction measures as reducing the number of customers, reduces perceived threat and thus reducing deviant shopping behavior. Besides there seem be other factors (e.g., perceived scarcity like found by Bentall et al. (2021) and now discussed) influencing shopping behavior thus maintaining a changed purchasing behavior. Since we did not control for the perceived effectiveness of measures (as reducing the number of customers) in our study and to the best of our knowledge there is no study investigating this factor in the context of the COVID-19 pandemic, we hesitate to add this aspect to our discussion.

**Reviewer 2:**

**To be honest, I found this paper very well written. Easy to read and to grasp. I do not have any major comment. I just took a couple of minor notes, while reading it:**

**The “perspective article” on what social and behavioural science can do to support pandemic response, publishes by Van Bavel et al. in Nature Human Behaviour can be a useful general reference for the Introduction.**

We thank the reviewer for raising our attention to the article by Van Bavel et al. In accordance with the reviewer´s suggestion we now refer to the article in the introduction:

“Understanding the causes for changes in consumers purchasing behavior under the COVID-19 pandemic is of high relevance for governments and policymakers, e.g., to avoid panic buying which in turn may cause shortage of important goods. As stated in a perspective article by Van Bavel and colleagues [20] there are several research topics relevant to the COVID-19 pandemic which have to be addressed by social and behavioral sciences. Fear is a central emotional response during a pandemic which shapes information processing (e.g., risk perception) and behavior (e.g., shopping behavior). […]”

**“As observed in animal studies, increased threat, i.e., increased probability of the predator entering the foraging area (as indicated by a visual cue) led to a reduction of the number of tokens being collected while participants stayed closer to the safe space, to increase chances of successful flight from predatory attack [10]”. My understanding is that there is something missing here, that is, the fact that the amount of money collected each time is greater. Perhaps they did not find this, simply because participants do not need money to actually survive. So, it’s possible that this setting is quite different from the setting under study in the paper.**

We agree with the reviewer that the experimental study by Bach et al. only provides limited comparability and therefore decided to remove it from the introduction. However, we still refer to the Bach study in our discussion section on future research as an example of experimental research on human foraging.

“More experimental studies should try to translate findings from animal experiments to human behavior to test if the certain behaviors are associated with different threat levels as reported in a study by Bach and colleagues […].”

**The result on gender differences in purchasing behaviour under threat could be related to the emerging literature on gender differences in COVID-19 preventative behaviours (Capraro & Barcelo, 2020; Coroiu et al. 2020; Haischer et al. 2020).**

We thank the reviewer for raising our attention to literature on gender differences in COVID-19 preventive behaviors. In accordance with the reviewer´s suggestion we now relate our finding to Capraro & Barcelo (2020):

“Although only included as control variable, we found that female sex was associated with a decrease in shopping frequency. This result could be interpreted in terms of more cautious behavior in female compared to male individuals. A study investigating the role of messaging and gender on intentions to wear nose-mouth-protection during the COVID-19 pandemic revealed that woman more than men intend to wear a face covering [40]. A mediating factor was the subjective likelihood to contract the disease, supporting our post-hoc hypothesis that women behave more cautious during the COVID-19 pandemic. Note that in our data female sex was associated with higher levels of *Perceived Threat of COVID-19*.”

**Reviewer 3:**

**1. Although I welcome a concise introduction, I still think that it would benefit from a deeper and more precise theoretical deduction of hypotheses on the association between anxiety psychopathology (intolerance of uncertainty, trait anxiety), media exposure and threat of COVID-19 or behavioral responses (e.g., for intolerance of uncertainty: Tull et al., 2020 [10.1016/j.janxdis.2020.102258]; Gertens et al., 2020 [10.1016/j.janxdis.2020.102258]). A short presentation of results of previous research on purchasing behavior and associated factors during the COVID-19 pandemic (e.g., Micalizzi et al., 2020 [10.1111/bjhp.12480] or quite new results from Bentall et al., 2021 [10.1371/journal.pone.0246339]), which also used a animal foreaging approach) would be likewise interesting.**

We thank the reviewer for raising our attention to further literature. We now give a more detailed review about vulnerability factors for anxiety disorders and the relation between media exposure and threat of COVID-19. Additionally, we cite the results obtained by Micallizi et al. (2020) in our introduction and give a detailed discussion on the results obtained by Bentall et al. (2021).

**Introduction:**

“A recent study investigated factors influencing stockpiling during the COVID-19 pandemic. Increased COVID-related worry (e.g., “I will become very ill.”; “I will not have access to food.”) was associated with stockpiling of more products indicating that negative affect like worries and anxiety influence shopping behavior [13].”

[…]

“Additionally, we were interested in the influence of other factors known to influence feelings of anxiety that thus might be associated with threat perception of COVID-19 and changes in purchasing behavior. It has been demonstrated that psychological vulnerability factors such as trait anxiety (i.e., the tendency to experience anxiety and perceive situations as threatening) and intolerance of uncertainty (i.e., the tendency of an individual to experience possible negative future events as unacceptable and threatening) increase the risk to fearfully respond to potentially negative or uncertain stimuli, events or situations such as those arising during the current pandemic. Both psychological factors have been linked with occurrence of anxiety-related disorders [14–16]. Initial evidence from the current COVID-19 pandemic revealed that trait-anxiety and intolerance of uncertainty are associated with higher levels of threat perception and fear of the coronavirus [17, 18]. Another relevant factor that has been discussed to increase fear and threat perception of COVID-19 via transmission of threat information is the level of exposure to media. Studies from current COVID-19 pandemic higlight the role of increased media exposure on elevated anxiety and stress responses as well as increased fear of COVID-19 under the COVID-19 pandemic [17, 19]. In the present study, we tested whether *Perceived* *Threat of COVID-19* explains changes in purchasing behavior beyond these factors.”

**Discussion:**

“Similar results were observed in another online survey which also used an animal foraging framework to explain changes in purchasing behavior [33]. In this study, perceived probability of getting an infection was positively associated with increased purchasing quantity. In contrast to the present study, the authors emphasized on threat due to scarcity which is not covered in our study. The moderate correlation between risk perception and *Perceived Threat of COVID-19* as found in our study suggests, that additional factors – as for instance threat of scarcity – might explain additional variance in perceived threat. According to Bentall and colleagues [33], perceived risk of infection is a factor influencing scarcity vulnerability. Future studies ought to include threat due to scarcity to test whether *Perceived Threat of COVID-19* remains a meaningful predictor for changes in purchasing behavior after controlling for threat due to scarcity. […]”

**Furthermore, I would like to see the authors name all hypotheses (hypotheses on the explanation of threat of COVID-19; risk for oneself/loved ones) and especially the direction of all studied hypotheses (e.g., expected direction of associations of intolerance of uncertainty/trait anxiety and purchasing behavior). This, then, also refers to the statement in the discussion, that some results have been unexpected (e.g., no association between being part of a risk group and purchasing behavior).**

In accordance with the reviewer´s recommendation we added a new section where we name all hypotheses.

“Our main hypotheses are that higher levels of *Perceived Threat of COVID-19* would be (1) associated with a reduction of purchasing frequency and (2) an increase in purchasing quantity per purchase. We also expected a positive correlation between *Perceived Threat of COVID-19* and an increase in purchasing quantity for individual products. In face of the known relevance of trait-anxiety and intolerance of uncertainty as risk factors for anxiety disorders and depression, we hypothesized that these constructs would be positively related to *Perceived Threat of COVID-19*. Additionally, we expected individuals with high vulnerability to develop anxiety disorders (high trait-anxiety, high intolerance of uncertainty) to show a decrease in shopping frequency and an increase in purchasing quantity per purchase. Besides we hypothesized that being part of a risk group for a severe course of an infection with COVID-19 or having regular contact with a high-risk person would be associated with higher levels of *Perceived Threat of COVID-19* and changes in shopping behavior as described above. A high extend of media exposure was also hypothesized to be positively associated with *Perceived Threat of COVID-19* and changes in purchasing patterns (increased purchasing quantity while reducing shopping frequency).”

**2. Additional analyses / hypotheses:**

**a. Explaining human purchasing behavior in the context of a pandemic situation with animal foraging models is an interesting approach. The cited study on human behavior under threat (Bach et al., 2014) (introduction) showed – in my opinion – only limited comparability to the cited animal studies, as the predator in this case did not endanger the “life” of people but “only” the collected rewards, which possibly overlaps more with an assumed shortage of goods in a pandemic situation. It, therefore, made me think, if the change of purchasing behavior in a pandemic situation could possibly be better or additionally explained by a presumed risk of shortage of goods than by the perceived threat of COVID-19 while buying. The authors do refer to this alternative hypothesis in the discussion section. I was still wondering whether the authors assessed and controlled for this aspect in the present study?**

We agree with the reviewer that the experimental study by Bach et al. (2014) only provides limited comparability and therefore decided to remove it from the introduction. However, we kept the reference in our discussion on future research as an example of experimental research on human foraging.

“More experimental studies should try to translate findings from animal experiments to human behavior to test whether certain behaviors are associated to different threat levels such as reported in a study by Bach and colleagues.”

We did not ask participants for a risk assessment of shortage of goods, although we agree that this seems to be a very important as shown by the results of Bentall et al. (2021) which we now discuss (see answer to point 1).

**b. Purchasing frequency and quantity are highly correlated in the present study. This absolutely makes sense, as one would assume that, if going less often grocery shopping, you would have to buy more when going grocery shopping. Did the authors perform regression analyses on purchasing quantity, which controlled for purchasing frequency?**

As stated by the reviewer, both variables were highly correlated. We performed a regression analyses to the model. Since Perceived Threat of COVID-19 correlated with both variables, this reduced the specific effect. However, Threat of COVID-19 still remained significant (see Table below).

“[…] Note that Perceived Threat of COVID-19 (*b* = .08, *p* = .026) remained significant predictor for purchasing quantity when controlling for purchasing frequency (see S4 Table).”

**Change in Purchasing Quantity controlling for change purchasing frequency.**

|  | **QuaMarch** | | |
| --- | --- | --- | --- |
| *Predictors* | *b* | *t-Statistic* | *p-value* |
| FreqMarch | **-0.56** | **-17.36** | **<0.001** |
| Sex | 0.03 | 0.41 | 0.681 |
| Age | -0.12 | -3.72 | **<0.001** |
| Education | 0.03 | 1.07 | 0.287 |
| Householdsize | 0.01 | 0.47 | 0.636 |
| Social Desirability | -0.02 | -0.58 | 0.561 |
| Perceived Threat of COVID-19 | **0.08** | **2.22** | **0.026** |
| IUS | 0.02 | 0.50 | 0.619 |
| STAI | -0.01 | -0.17 | 0.864 |
| Media Exposure | 0.06 | 1.85 | 0.065 |
| Risk Perception | 0.06 | 1.72 | 0.086 |
| Observations | 678 | | |
| R^2^ / R^2^adjusted | 0.405 / 0.395 | | |

**c. Explained variance of purchasing frequency and quantity could be considered as rather small in all analyses. Reflecting on that, I asked myself, which other variables could contribute to purchasing behavior in a pandemic situation. Additionally, to the assumption already mentioned in a.), I still wondered, if their could also have been effects of the amount of staying-at-home-time on purchasing quantity (e.g., no canteen during lunch break, no visits of restaurants). Did the authors ask the participants to rate the amount of home-prepared meals? If not so, I still would appreciate a short reflection on the “rather low” explained variance and other possibly influencing factors in the discussion.**

We thank the reviewer for raising our attention to this important issue. We did not ask participants to rate the amount of home-prepared meals. In accordance with the reviewer´s suggestion, we now discuss additional potential influences on shopping behavior.

“The explained variance for change in purchasing frequency and purchasing quantity was rather small, indicating that additional factors were associated with a change in purchasing behavior. Recent studies indicate that e.g., right political affiliation [Micalizzi+2020, Bentall+2020], the extend of engaging in social distancing [Micalizzi+2020], and higher levels of paranoia [Bentall+2020] are associated with more stockpiling.”

**d. The authors excluded people, who, e.g., have been infected with COVID-19 or who did not buy their purchases before the COVID-19 pandemic, which can be considered as plausible. I am still interested if the authors did analyze, even on an exploratory purpose, the associations between perceived threat of COVID-19 and the decision to not make one’s own purchases (n = 150) or to exclusevily buy online (n = 62). One could assume that especially the latter group would show higher levels of perceived threat of COVID-19.**

We thank the reviewer for raising our attention to this interesting aspect. Please, note that questions regarding shopping behavior (online-purchasing, doing one´s own purchases) were answered for pre-pandemic purchasing strategy as well as for purchasing during the pandemic.

We checked the reviewer´s hypothesis by creating subsamples of participants who changed their purchasing behavior, e.g., did not purchase online before the outbreak of COVID-19, but did so after the outbreak (*N =* 49), and compared this group to the main analysis sample (N= 813). The same logic was applied regarding participants indicating doing their own purchases before the outbreak, but not anymore after (*N =* 23).

Interestingly, participants who changed to online buying, felt less threatened by the COVID-19 pandemic (*t*(860) = 3.19, *p* = .001) than the main group, suggesting that online buying possibly might be an effective strategy to mitigate feelings of threat. Similarly, participants, who terminated making their own purchases also reported significantly lower perceived threat than the main group (*t*(834), *p* = .03).

Since the sample sizes of both subgroups were rather small and the study not designed to explicitly test this question, we hesitate to report these post-hoc findings in the manuscript.

**e. The separate calculation of regression models in the total sample and in groups reporting a decrease or stability of purchase frequency and in groups reporting an increase or stability of purchase quantity seems to be plausible considering the proposed hypotheses. I highly welcome the additional reporting of results of the full range scale as a supplementary table. I, nevertheless, asked myself, if the authors also have calculated the “total regression model” (likewise to Table 3) of the full range scale of purchasing frequency and quantity and regression models on the full range scale of purchasing quantity for individual products. Did the reported results and conclusions stay stable?**

In accordance with the reviewer suggestion, we calculated and now report the total regression model.

The effect of *Perceived Threat of COVID-19* did stay stable for “Non-perishable food” and “Hygiene Products”, but not for “Fresh Food”. As previously done for the full range scale, we now added these analyses as a supplement to this manuscript and give a reference in the results section (see **S6 Table** and Table below).

**Full Range Scale individual products.**

|  | **Non-perishable Food** | | | **Hygiene Products** | | | **Fresh Food** | | |
| --- | --- | --- | --- | --- | --- | --- | --- | --- | --- |
| *Predictors* | *b* | *t* | *p* | *b* | *t* | *p* | *b* | *t* | *p* |
| Sex | 0.07 | 1.13 | 0.261 | 0.08 | 1.27 | 0.204 | 0.09 | 1.42 | 0.156 |
| Age | 0.00 | 0.35 | 0.729 | -0.00 | -1.43 | 0.153 | -0.00 | -0.47 | 0.640 |
| Education | 0.04 | 1.22 | 0.223 | **0.07** | **2.36** | **0.018** | 0.01 | 0.47 | 0.639 |
| Household Size | 0.02 | 1.08 | 0.281 | 0.00 | 0.05 | 0.964 | 0.01 | 0.43 | 0.668 |
| Social Desirability | **-0.03** | **-2.23** | **0.026** | -0.02 | -1.64 | 0.100 | -0.01 | -0.70 | 0.482 |
| Risk Group (self) | 0.05 | 0.73 | 0.463 | -0.06 | -1.02 | 0.308 | -0.02 | -0.37 | 0.712 |
| Risk Group (others) | -0.04 | -0.65 | 0.518 | 0.02 | 0.37 | 0.714 | 0.05 | 0.96 | 0.338 |
| Media Exposure | 0.07 | 1.85 | 0.065 | **0.10** | **2.57** | **0.010** | 0.03 | 0.75 | 0.454 |
| Threat COVID-19 | **0.10** | **4.26** | **<0.001** | **0.07** | **3.43** | **0.001** | 0.02 | 1.09 | 0.275 |
| Risk Perception | **0.00** | **2.11** | **0.036** | 0.00 | 1.46 | 0.145 | 0.00 | 0.44 | 0.662 |
| IUS | **0.01** | **2.97** | **0.003** | **0.01** | **3.36** | **0.001** | 0.00 | 1.67 | 0.095 |
| Observations | 810 | | | 813 | | | 794 | | |
| R^2^ / R^2^adjusted | 0.096 / 0.083 | | | 0.096 / 0.083 | | | 0.017 / 0.003 | | |

**3. Data analysis:
a. Model assumptions / Robustness of results:
i. I was wondering if the authors did check for regression model assumptions? Detected medium correlations between some predictor variables (S1 Table 1, Table 2) could, e.g., indicate some multicollinearity of predictor variables.
ii. Nevertheless, in order to gain more robust results, did the authors consider using bootstrapping? If not so, I would still suggest adding confidence intervals and standard errors for all regression estimates.**

We thank the reviewer for raising our attention to this important issue. Multicollinearity was not a problem in our models. All variance inflation factors (VIF) were smaller than 2 (values above 5 are considered as problematic). We have now added a comment on this issue in the results section. Since the assumption of normal distributed residuals is not fully fulfilled, we followed the recommendation of using bootstrapping. The obtained results are highly comparable. In accordance with the reviewer’s suggestion, we now report confidence intervals based on bootstrapping (r = 2000) in addition to the parametric tests.

“We checked for multicollinearity using the variance inflation factor (VIF). All VIFs were smaller than two and thus considered unproblematic [30]. Since the dependent variables (purchasing frequency and purchasing quantity) were not normally distributed, we decided to additionally report confidence intervals (95% CI) based on bootstrapping [31] to bypass the assumptions for multiple linear regression. 2000 samples were generated to obtain an empirical distribution (using the boot.ci-function from the r package “boot”). Note that these results were highly comparable to the results of the parametric test. […]”

**b. Data level:
i. As the outcomes “purchasing frequency” and “purchasing quantity” rather are ordinal variables, I was wondering if the authors considered using non-parametric tests for calculation of differences of central tendencies (e.g., Wilcoxon) or for performing regression analyses (ordinal regression)? Regarding the paired samples t-tests, I would highly recommend to compute and report effect size measures (e.g., Cohen’s d)**

We agree with the reviewer that our outcomes can be considered as ordinal variables. We have therefore reanalyzed the data using an ordinal logistic regression (see table below) The analysis revealed the same pattern of results and is now attached as supplementary information (**S3 Appendix**). Since the cited literature about changes in shopping behavior (e.g, Garbe et al. 2020, Bentall et al. 2021) uses a multiple linear regression account, we would prefer to stick with the parametric solution for better comparability, but now provide the alternative analysis as a supplement. In accordance with the reviewer´s suggestion we now report Cohen´s d for the reported t-tests.

|  | **Change in Purchasing Frequency** | | | **Change in Purchasing Quantity** | | |
| --- | --- | --- | --- | --- | --- | --- |
| *Predictors* | *Odds Ratios* | *CI* | *p-value* | *Odds Ratios* | *CI* | *p-value* |
| Sex | **1.59** | **1.11 – 2.28** | **0.012** | 0.75 | 0.51 – 1.08 | 0.127 |
| Age | 0.96 | 0.83 – 1.12 | 0.625 | 0.77 | 0.65 – 0.90 | **0.002** |
| Educational Level | **0.78** | **0.67 – 0.90** | **0.001** | **1.28** | **1.10 – 1.50** | **0.002** |
| Household Size | 0.99 | 0.86 – 1.15 | 0.882 | 1.00 | 0.85 – 1.16 | 0.981 |
| Social Desirability Bias | 1.01 | 0.87 – 1.17 | 0.903 | 0.92 | 0.78 – 1.07 | 0.270 |
| Threat of COVID-19 | **0.59** | **0.49 – 0.70** | **<0.001** | **1.55** | **1.29 – 1.87** | **<0.001** |
| IUS | 1.13 | 0.94 – 1.36 | 0.212 | 1.01 | 0.84 – 1.23 | 0.881 |
| Trait-Anxiety | 0.92 | 0.77 – 1.10 | 0.373 | 1.02 | 0.84 – 1.24 | 0.821 |
| Media Exposure | **0.79** | **0.67 – 0.92** | **0.004** | **1.36** | **1.14 – 1.61** | **0.001** |
| Perceived Risk | **0.81** | **0.70 – 0.94** | **0.006** | **1.27** | **1.08 – 1.49** | **0.003** |
| Observations | 678 | | | 678 | | |
| R^2^ Nagelkerke | 0.168 | | | 0.165 | | |

**Overall Model: Prediction of change in purchasing behavior (frequency/quantity) using Ordinal Logistic Regression. Compare with Table 3 in the manuscript.**

**ii. Correlational analyses: Again, considering the different data structure of the reported variables, I was asking myself if the authors took this into account when computing correlational analyses (e.g., Spearman Rho)?**

As stated above (i.), we agree with the reviewer that our outcomes can be considered as ordinal variables. We recalculated all correlations as spearman rank coefficient revealing a very similar pattern of results. Since most of the other variables would be considered as interval scaled variables (e.g., Threat of COVID-19, IUS, STAI which are mean, respectively, sum scores and thus treated as interval scaled variables) we would like to use one common coefficient for all results in the manuscript, but now also provide these non-parametric results (spearman rank coefficients) as a supplementary information (see S3 Appendix).

We give a reference to the supplement in the method section:

“Additionally, we report non-parametric analyses (e.g., ordinal logistic regression) for the main findings as supplementary information (see S3 Appendix), again, showing highly comparable results. […]”

**c. The authors have not commented on the handling of missing data yet. Has there been missing data and how have the authors proceeded with missing data?**

We apologize for the misunderstanding. Data analysis was restricted to participants who gave an answer to every question. Participants who did not finish the questionnaire were excluded from further analysis. The only “missing data” occurred for the calculation of individual products since there was an option to indicate not buying a product at all. Therefore, the sample size varies for the individual products when using the full range scale (see S6 Table). We extended the section “Participants” as follows:

“In total 1074 individuals completed the online questionnaire and gave an answer to every question. Participants who did not finish the questionnaire were excluded. Data analysis was further limited to […].”

**4. Minor comments:
a. Abstract: I would propose to add exact statistics (p-values, b) for the main reported effects (associations between threat of COVID-19 and purchasing frequency and quantity, associations between intolerance of uncertainty and extend of media exposure and threat of COVID-19).**

We added exact statistics for the main effects in the abstract.

**b. Paragraph 2 on page 3: The sentence “Based on a theoretical framework […]” sounds more like a summary of an aim or like a introduction to the presentation of hypotheses. Therefore, I would suggest to move this statement and either include it as an introduction to your hypotheses or add it to the discussion.**

We thank the reviewer for raising our attention to this point. In accordance with the reviewer´s suggestion we use this statement as introduction for our hypotheses.

“Understanding the causes for changes in consumers purchasing behavior under the COVID-19 pandemic is of high relevance for governments and policymakers, e.g., to avoid panic buying which in turn may cause shortage of important goods. As stated in a perspective article by Van Bavel and colleagues [20] there are several research topics relevant to the COVID-19 pandemic which have to be addressed by social and behavioral sciences. Fear is a central emotional response during a pandemic which shapes information processing (e.g., risk perception) and behavior (e.g., shopping behavior). Based on a theoretical framework derived from rodent foraging behavior under threat, we examined the role of perceived threat originating from the present COVID-19 pandemic situation in predicting changes in purchasing patterns of groceries and hygiene products in an online questionnaire study. Our main hypotheses are that higher levels of perceived threat of COVID-19 would be (1) associated with a reduction of purchasing frequency and (2) an increase in purchasing quantity per purchase. […]”

**c. In the first paragraph of the method section (pages 4-5), the authors refer to the implementation of public health measures, and to an increase of risk communication and sales figures in Germany. I would consider it helpful to include references and examples for these statements.**

We included references for the implementation of public health measures and risk communication.

“The study was conducted from April 23^rd^ to May 18^th^, 2020. In this time window the total amount of confirmed COVID-19 cases in Germany had reached 175.896. The implementation of public health measures by the German federal states started in March 2020 (e.g., prohibition to meet with others in public places, closure on non-essential shops, or closure of kindergartens or daycare institutions [21] while risk communication increased in the media, e.g., daily report of case numbers or information that infection with COVID-19 may cause a life-threatening disease and recommendations on how to avoid infection [19]. In effect, for March 2020 massive increases in sales figures were reported [3]. […]”

**d. As perceived threat of COVID-19 serves as the main predictor in the present study, I would like to see the authors name all six items which constitute the mean score. I might have missed it, but did the authors name the scaling of the rating scales of perceived threat of COVID-19 in the measures section?**

We thank the author for raising our attention to this point. We now name all six all variables in the method section:

“*Perceived Threat of COVID-19*: *Perceived Threat of COVID-19* was measured using six semantic differential seven-point rating scales. The six items were introduced with “*The novel coronavirus is for me ... .”* following two oppositely poled adjectives, (“concerning” vs. “not concerning”, “frightening” vs. “not frightening”, “something I am thinking about all the time” vs. “something I am not thinking about all the time”, “something I feel helpless about” vs. “something I can actively do something about”, “burdensome” vs. “not burdensome”, “close” vs. “far away”). […].”

**e. As I assume that the authors used the German versions of the presented measurement instruments, I would suggest adding the respective references and reporting the internal consistencies from “German” validation studies. Additionally, I am missing a comment on validity of used instruments in previous research.**

We thank the author for raising our attention to this point. We know added reference for the German version of both scales. In the revisited introduction we now refer to literature indicating the validity of the STAI and IUS (see also answer to point 1).

*“Intolerance of Uncertainty Scale*: We used the 12-item short version of the *Intolerance of Uncertainty Scale* which maps the tendency of an individual to experience possible negative future events as unacceptable and threatening (e.g., “Unforeseen events upset me greatly.”) and is associated with worry, state anxiety and related to anxiety pathologies [22]. The reported internal consistency of the short version is Cronbach´s *α* = .91. The internal consistency in this sample was good (Cronbach´s *α* = .87). A German validation study reported similar results (Cronbach´s *α* = .90) and reported intolerance of uncertainty to be predictive for worrying [23].

*State Trait Anxiety Inventory*: We used the trait portion of the *State Trait Anxiety Inventory* (A-Trait, e.g., “I worry too much over something that really doesn´t matter.”) which consists of 20 items. The internal consistency in this sample reached an excellent value of Cronbach´s *α* = .94**.** The reported Cronbach´s *α* for the A-Trait lies between .86 - .95 [24], for the German version Cronbach´s *α* = .90 [25].”

**f. To increase the reading flow, please consider reviewing the manuscript on consistent naming, spelling, and highlighting (e.g., italics) of central concepts (e.g., trait-anxiety vs. trait anxiety, behaviour vs. behavior, COVID-19-Threat vs. threat of COVID-19, sex vs. gender)**

In accordance with the reviewer’s suggestion we now use a consistent spelling and highlighting of central concepts.

**g. I would like the authors to review all tables on consistent and “correct” adding of zeros before the decimal point (b and p-values)**

We thank the reviewer for raising our attention to this point. We checked all tables on consistency regarding the reported statistics.

**h. Table 2 and 3 report results on both purchasing frequency and quantity. As stated by the authors before, they have used a restricted sample for the analyses reported in table 2 and 3. In the footnotes of the tables, N is, nevertheless, stated as being the same. I would like the authors to review the footnotes of the tables 2 and 3.**

We apologize for the misunderstanding. The analyses reported in Table 2 and 3 were conducted on the same restricted sample. The analysis in Table 2 reports the specific effects of adding one predictor to the baseline model. Table 3 reports the specific effects when entering all significant results from the “Table 2”-Analysis in one step. In the supplement we report the latter analysis for the full range scale. For the sake of clarity, we now added a statement to the footnote of the tables

**Footnote Table 2:**

“Multiple linear regression performed on the restricted sample (*N* = 678). Table reveals the specific effect for a single predictor above baseline model. Significant regression weights (*p < .05*) of the multiple regression analysis are printed in bold. All continuous variables were included as z-standardized variables. Dichotomous Variables: Coding for sex: female = 0, male = 1; coding for being part a risk group for a severe COVID-19 disease course: no = 0, yes = 1. *Perceived Threat of COVID-19* was the best predictor for change in purchasing frequency (*R²adj.* = .12) and change in purchasing quantity (*R²adj.* = .11). *Suppressor Effect: Variable did not reveal a significant bivariate correlation with the criteria.”

**Footnote Table 3:**

“Multiple linear regression on the restricted sample (*N* = 678). In this analysis all predictors adding significant variance above the baseline model were entered in one step. Significant regression weights (*p* < .05) of the multiple regression analysis are printed in bold. All continuous variables were included as z-standardized variables. Coding for sex: female = 0, male = 1.”

**i. Although the coding of the educational level is explained in the methods section, an additional footnote on the coding of educational level in Table 1 would be beneficial.**

We added an additional footnote on the coding of educational level.

Finally, we would again like to thank the reviewers for their thoughtful comments and suggestions. We hope that we have addressed all the remaining points and we also hope that you and the reviewers will find the manuscript suitable for publication in PLOS ONE. For correspondence, please, contact Sebastian Schmidt. Thank you for giving this paper your consideration.

Yours sincerely,

Sebastian Schmidt
